# Proteomic analysis of infiltrating neutrophils from rheumatoid arthritis synovial fluid and their contribution to protein carbamylation

**DOI:** 10.3389/fimmu.2025.1563426

**Published:** 2025-04-09

**Authors:** Xueting Chen, Rong Du, Pan Wang, WenLin Qiu, Lu Chen, Jian Wan, Hui Qiu, Li Xiong, Kutty Selva Nandakumar, Rikard Holmdahl, Hui Geng

**Affiliations:** ^1^ Hubei Key Laboratory of Genetic Regulation and Integrative Biology, School of Life Sciences, Central China Normal University, Wuhan, China; ^2^ Department of Rheumatology, Union Hospital, Tongji Medical College, Huazhong University of Science and Technology, Wuhan, China; ^3^ Department of Radiation and Medical Oncology, Zhongnan Hospital of Wuhan University, Wuhan, China; ^4^ Department of Environmental and Biosciences, School of Business, Innovation and Sustainability, Halmstad University, Halmstad, Sweden; ^5^ Medical Inflammation Research, Division of Immunology, Department of Medical Biochemistry and Biophysics, Karolinska Institute, Stockholm, Sweden; ^6^ National-Local Joint Engineering Research Center of Biodiagnosis and Biotherapy, The Second Affiliated Hospital of Xi’an Jiaotong University, Xi’an, Shanxi, China

**Keywords:** carbamylation, myeloperoxidase, neutrophil, synovial fluid, rheumatoid arthritis

## Abstract

**Introduction:**

Carbamylated proteins and dysregulated neutrophils are implicated in rheumatoid arthritis (RA) pathogenesis. Herein, we characterized the neutrophils present in RA synovial fluid (SF) using proteomic techniques and evaluated their contribution to protein carbamylation.

**Methods:**

RA-SF neutrophil proteomic profile and SF proteome signature were investigated using high-resolution mass spectrometry. Carbamylated proteins and the degree of protein carbamylation were evaluated by mass spectrometric analysis. ELISA and chemiluminescence kits were used to examine myeloperoxidase (MPO) activity, and hydrogen peroxide (H_2_O_2_) generation.

**Results:**

SF neutrophils exhibited a shift in proteomic cargo with up-regulated proteins involved in defense responses, neutrophil degranulation, and reactive oxygen species metabolic processes, while proteins down-regulated were associated with megakaryocyte differentiation, leukocyte migration, and integrin-mediated signaling pathway. Elevated levels of neutrophil-derived proteins were detected in RA-SF. In addition, we specifically identified many carbamylated proteins and observed an increased frequency of protein carbamylation in RA-SF samples. Functionally, neutrophils from RA-SF showed a significantly increased level of MPO release and HH_2_O_2_ generation. Moreover, MPO activity was higher in RA-SF than in autologous blood samples, which correlated well with the degree of protein carbamylation in RA-SF.

**Discussion:**

Synovial neutrophils were found to be activated and increased releasing protein cargo, including MPO and ROS, into the synovial fluid. Presence of many carbamylated proteins in RA-SF and an increased MPO activity showed a strong correlation to the degree of protein carbamylation, suggesting neutrophil-derived MPO in promoting generation of aberrantly carbamylated proteins.

## 1 Introduction

Rheumatoid arthritis (RA) is a common autoimmune disease with the hallmark of multiple autoantibodies to abnormal post-translational modifications (PTM) of proteins. In addition to the citrullination of protein arginine residues, irreversible N-ϵ carbamylation of lysine residues attracts increasing attention as an important PTM as a relevant antigen in RA patients (1–3). About 50% of RA individuals have detectable circulating antibodies to carbamylated proteins (anti-CarP), and the presence of anti-CarP antibodies occurs before the onset of disease and has been suggested to be associated with poor prognosis implicating that anti-CarP interact with RA pathogenesis (1, 4, 5).

Carbamylation of proteins is formed by two distinct biochemical pathways. One results from the binding of cyanate (CNO^−^) derived from urea dissociation, and the other involves myeloperoxidase (MPO) oxidized thiocyanate (SCN^−^) to cyanate. However, normal plasma urea concentration is too low to induce protein carbamylation, and an aberrant carbamylation was only detected in uremia secondary to chronic renal insufficiency (6, 7). Carbamylation *in vivo* is normally formed due to neutrophilic inflammation, and is likely related to MPO and reactive oxygen species (ROS) from neutrophils (8, 9). MPO uses H_2_O_2_ and thiocyanate ions (SCN^−^) as co-substrates to produce cyanate (OCN^−^), promoting protein carbamylation at inflammation sites (8, 9). Although Cl^–^ is the most abundant MPO substrate in plasma, SCN^−^ is a more favorite substrate than Cl^−^ for MPO activity (730-fold greater), therefore, cyanate is a major product of MPO (10). Actually, the presence of HOCl promotes the formation of cyanate from cyanide under MPO catalysis (8, 11). Consistent with these findings, a previous study reported that MPO specifically induces protein carbamylation rather than chlorination in human atherosclerotic lesions (12). Hence, leukocyte-derived MPO activity, and reactive oxygen species (ROS) production might be the major pathways that foster protein carbamylation occurring at inflammatory sites.

The presence of carbamylated autoantigens in the joints of RA patients could be due to the progressing chronic inflammation in arthrodial joints. Advances in mass-spectrometry permit the identification of such carbamylated proteins from cartilage and synovial tissue of RA patients (13, 14). However, the mechanisms leading to the carbamylation of proteins in RA joints still need to be explored.

Neutrophils are the most abundant leukocytes in the inflamed synovial fluid, and growing evidence supports the critical role of dysregulated neutrophils in RA pathogenesis (15, 16). The importance of neutrophils in mediating PTM dysregulation is well recognized. As described previously, synovial neutrophils are important immune cell sources of citrullinated proteins by releasing active PAD enzymes via neutrophil extracellular trap (NET) formation (17, 18). MPO is the major neutrophil protein, and a key component and critical enzyme released by neutrophils through degranulation or NET formation (19, 20). The effects of MPO on RA have gained attention recently and elevated MPO levels are associated with RA severity (21). Enzymatically active MPO level is up-regulated in RA joints, and oxidant formation by MPO contributes to disease development (22, 23).

Herein, we examine RA-SF neutrophil proteomic profile, the extent of protein carbamylation and neutrophil’s contribution in promoting protein carbamylation within the inflamed joints.

## 2 Materials and methods

### 2.1 Patient selection

Synovial fluid and blood samples were obtained from RA patients attending the Department of Rheumatology, Union Hospital, Tongji Medical College, China. RA patients fulfilled the 1987 ACR/2010 ELAR criteria, and patient characteristics are described in **Supplementary Table S1**. Synovial fluid and blood samples were collected from RA patients (n = 27) when knee joint arthrocentesis was done. Blood samples from the age- and gender-matched healthy controls (HC, n = 24) were collected from individuals recruited by advertisement. All participants signed informed consent. This study was approved by The Ethics Committee of the Union hospital, Tongji medical college (No [2021]. IEC(249)).

### 2.2 Isolation of neutrophils from SF and blood

SF and blood samples were collected into lithium-heparin tubes, and neutrophils were isolated within 30 min. SF was centrifuged at 400 g for 10 min to pellet the cells. In case of erythrocyte contamination, cells were re-suspended in phosphate-buffered saline (PBS) with 10 mM EDTA at a concentration of 1x10^6^/ml and mixed with hypotonic lysis buffer (GE Healthcare) for erythrocyte depletion. Neutrophils were further isolated from SF by Ficoll-Paque (GE Healthcare) according to the manufacturer’s protocol. Peripheral blood was separated by Ficoll-Paque, erythrocytes in the pellet were lysed by hypotonic solution, and neutrophils were purified by negative selection with EasySep™ direct human neutrophil isolation kit (STEMCELL). The collected cells were used for cytological identification, cell counting, *in vitro* stimulation experiments, or cryopreserved at -80°C until used for MS analysis. Neutrophil purity from SF and blood was > 96% (**Supplementary Figure S1**).

### 2.3 Preparation of cell-free SF and plasma

Following centrifugation to remove cells as described above, the SF supernatant and plasma were transferred into a new tube and centrifuged at 2000 g for 10 min to remove any remaining cells or debris. The freshly collected cell-free SF and plasma were used for detection of MPO activity or frozen at -80°C until used.

### 2.4 Purification of albumin from the SF and plasma

Albumin was purified from the SF or plasma using a Pierce™ Albumin Depletion Kit (Thermofisher) following the manufacturer’s protocol. Bound albumin was eluted by adding 1.5 M NaCl. The purity of albumin was checked by SDS-PAGE and Coomassie blue stain (**Supplementary Figure S2**). Purified albumin was used for MS analyses to detect the degree of protein carbamylation.

### 2.5 Preparation of peptide samples

Neutrophil pellets were dissolved in 50 µl lysis buffer containing 0.1% ProteaseMax (Promega) and sonicated for 15 min at 4°C. Lysate protein concentration was determined by bicinchoninic acid (BCA) assay. For cell-free SF and plasma peptide preparation, a volume corresponding to 100 μg protein was diluted in PBS with 0.1% ProteaseMax to a final volume of 50 µl. Twenty micrograms of purified albumin were dissolved in 50 μl of PBS and subjected to tryptic peptide digestion. Tryptic peptides were obtained by filter-aided sample preparation method (FASP) (24). Briefly, protein samples were transferred to a 10 kDa cutoff spin-filter (Millipore), reduced with 60 mM dithiothreitol (DTT) in 25 mM NH_4_HCO_3_ at 56°C for 60 min, blocked with 130 mM iodoacetamide (IAA) at room temp for 45 min in the dark, then digested overnight with trypsin and peptide at a ratio of 1:100 (wt/wt, Promega) for overnight at 37°C. The peptides were obtained by centrifuging the spin filters and desalted with a C18 pipet tip (Millipore), lyophilized with SpeedVac and kept at -20°C until used for MS analysis. For each sample, three technical replicates were investigated by mass spectrometry (MS) analysis.

### 2.6 MS data acquisition

The peptides were analyzed on Thermo Easy-nLC 1200 coupled to an Orbitrap Fusion Tribrid mass spectrometer (Thermo Scientific) equipped with a nano-electrospray ion source. Peptides were loaded on Thermo Acclaim Pepmap precolumn followed by an Acclaim Pepmap Easyspray analytical column. Separation was performed by binary gradient buffer A (4.9% acetonitrile, 0.1% formic acid) plus buffer B (90% acetonitrile, 0.1% formic acid). Peptides were loaded at a flow rate of 300 nL/min with 5% buffer B, equilibrated with 5% buffer B (5 min), eluted by 5-25% B (54 min), 25-40% B (25 min) with a hold (5 min), followed by 40-80% B (5 min) and 80-100% B (5 min). The electrospray voltage was set to 2.0 kV. The MS instrument was operated in data-dependent acquisition mode with the following settings: Full MS scan at a resolution of 70,000, with automatic gain control (AGC) target value 3e6; MS2-scan at a resolution of 17,500, with AGC target 5e4; the maximum injection time was 50 ms, and NCE step set to 27. The scan range was set at a resolution of 350-2,000 m/z, and the 20 most abundant ions were chosen for analysis.

### 2.7 Quantitative proteomics analysis

Raw MS data were searched by MaxQuant software (version 1.6.17.0) against the human proteome database (204,957 sequences). Search parameters for precursor and fragment tolerances were 5 and 20 ppm, respectively. A peptide false discovery rate (FDR) was set to 1%. Carbamidomethylation (Cys) was set as fixed modification, and carbamylation (K), acetylation (K or protein N-terminus), oxidation (M), and deamidation (NQ) were set as variable modifications. With trypsin, two missed cleavages were allowed, and the minimum peptide length was six amino acids. Relative protein quantities were calculated by summing up the unique peptide peak areas of each protein using the LFQ (label-free quantitation) feature (25). The LFQ values were then loaded to Perseus (v2.1.1.0) and transformed to log2 scale. The three technical replicates per experimental condition were grouped and averaged based on the median, and proteins were filtered for at least two valid values in one of the experimental groups. The missing values were replaced by a normal distribution (width = 0.3, shift = 1.8), assuming these proteins were close to the detection limit.

### 2.8 Quantitation of carbamylation

To distinguish between K-carbamylation and acetylation, we used MaxQuant software and performed searches with 5 and 20 ppm mass tolerance for precursor and fragment, respectively. This process reduces the chances of mismatching carbamylation and acetylation. The intensities of K-carbamylated peptides, and the corresponding parent peptides without modifications, were extracted by MaxQuant and Xcalibur software (version 2.2.0). Quantification of carbamylation was calculated as intensities of carbamylated peptides, divided by the sum of intensities of identification of parent peptide, independent of modifications.

### 2.9 Measurement of MPO activity

MPO activity in SF and plasma was determined using Innozyme activity assay kit (Merck-Millipore) by following the manufacturer’s protocol. To measure MPO activity in the neutrophil supernatant, freshly isolated neutrophils were re-suspended in RPMI 1640 media containing 2 mM L-glutamine, 25 mM HEPES at a concentration of 5x10^6^/ml, seeded in a MaxiSorp 96-well plate (Nunc), and incubated in a 5% CO_2_ incubator at 37°C for 60 min. Neutrophils were subsequently treated with or without 1 μM formylated Met-Leu-Phe tripeptide (fMLF, Sigma-Aldrich) for 2 h. Cell supernatants were collected and used for measuring MPO activity.

### 2.10 Measurement of H_2_O_2_ production

H_2_O_2_ production was measured using Luminol-enhanced chemiluminescence, as described previously (26). Neutrophils isolated from SF and blood were suspended at 1x10^6^ cells/ml in Hanks’s balanced salt solution and equilibrated at 37°C for 5 min. The assay mixture contained 10 μg/ml luminol (5-amino-2,3-dihydro-1,4-phatalazinedione; Sigma) and 5 U horseradish peroxidase (Roche), with or without 1 μM fMLF tripeptide. The light emission was recorded after adding the stimulus mixture for 20 min with an LB96V plater luminometer reader (Berthold, Germany).

### 2.11 Statistical analysis

Data were analyzed using GraphPad Prism (version 9.0) and SPSS (version 23.0) software. Paired samples were analyzed with Wilcoxon’s matched-pairs signed rank test, and the individual statistical tests were analyzed using Mann-Whitney U test. Spearman’s rho test was used for analyzing MPO activity and the degree of carbamylation. The p values < 0.05 were considered as significant.

## 3 Results

### 3.1 Synovial fluid neutrophils from RA patients exhibit distinct protein profiles compared to their blood neutrophils

**Figure 1:**
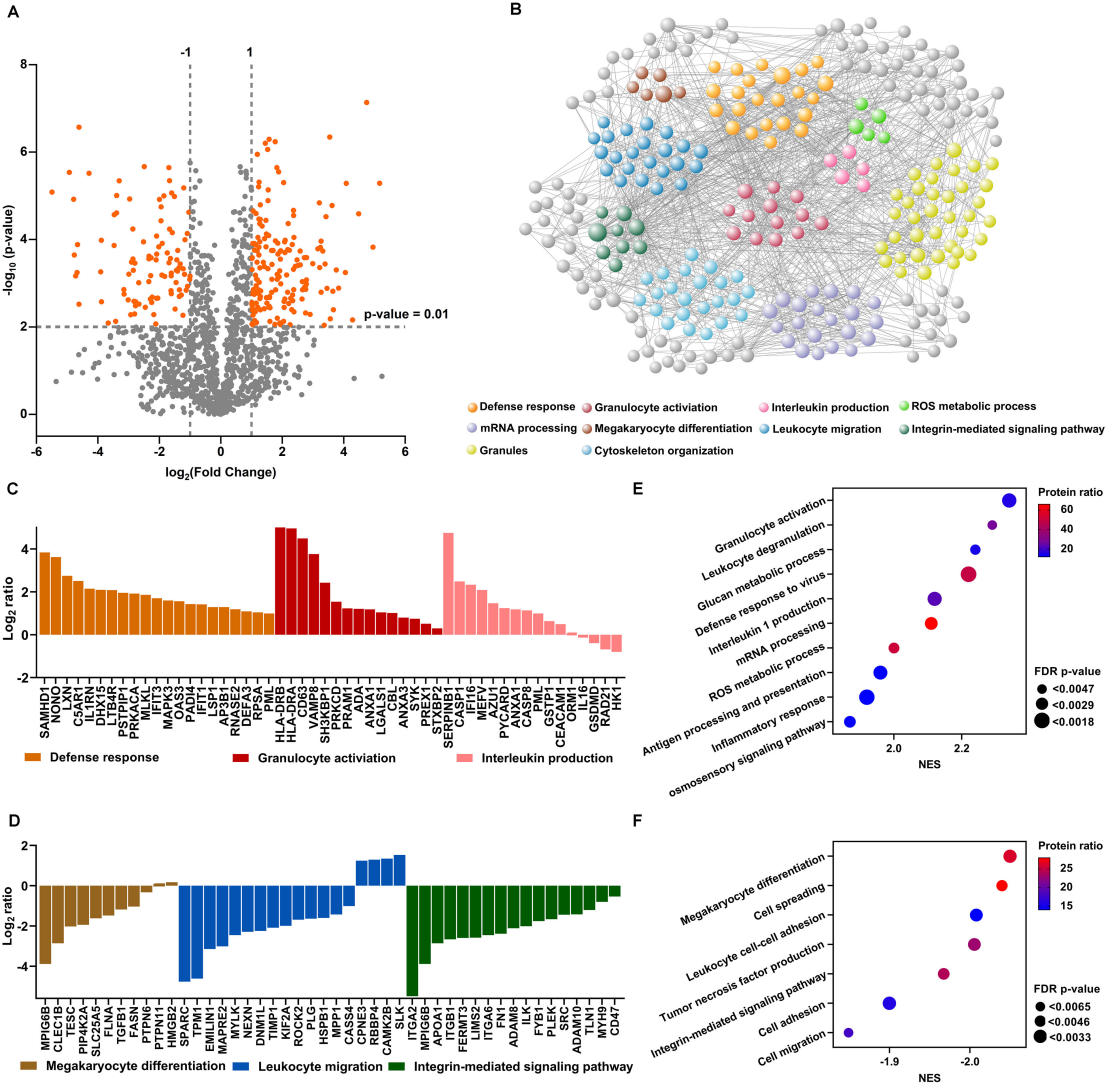
Differential protein expression in neutrophils from RA-SF versus blood. **(A)** Volcano plots illustrating differential protein abundance between neutrophils from RA-SF versus blood. Vertical and gray lines indicate the limit where proteins passed a significance value. **(B)** Interaction network of significant proteins from neutrophils from RA-SF versus autologous blood, developed using the online platform STRING. **(C, D)** Histogram showing log2 fold changes of individual protein clusters indicated in **(B, E, F)** Gene set enrichment analysis of the proteome of neutrophils from RA-SF versus blood. Normalized enrichment scores (NES) were displayed in different sizes to reveal the FDR-adjusted p-values using different colors to indicate the protein ratio within the indicated pathway.

To investigate proteomic profile shifts by neutrophils migrating into inflammatory joints, we performed high-resolution mass spectrometric analysis on neutrophils from RA-SF and blood from 4 RA patients with active established disease. We identified 2728 proteins with a false discovery rate (FDR) of less than 1% and quantified 2313 proteins (≥2 unique peptides for each protein). Label-free quantification (LFQ) analyses revealed an abundance of 182 significantly up-regulated proteins and 101 down-regulated proteins in the neutrophils from RA-SF compared to autologous blood (**Figure 1A**, **Supplementary Table S2**). Among them, the most frequent interactions involved belong to defense responses, granulocyte activation, interleukin production, ROS metabolic processes, mRNA processing, megakaryocyte differentiation, leukocyte migration, granules component, integrin mediated signaling pathway, and cytoskeleton organization (**Figure 1B**, **Supplementary Table S3**).

The top highly-expressed proteins identified are associated with defense responses, granulocyte activation, and interleukin production (**Figure 1C**). In particular, cell activation proteins like HLA-DRB, HLA-DRA, and CD63 (lysosome-associated membrane proteins-3, LAMP-3) were among the most over-expressed proteins (36.05, 30.99, and 22.45-fold higher in neutrophils from RA-SF versus autologous blood, **Supplementary Table S2**). HLA-DRB, HLA-DRA, and CD63 up-regulation are hallmark features of neutrophil activation. Conversely, proteins involved in megakaryocyte differentiation, leukocyte migration, and integrin-mediated signaling pathway were mostly under-expressed in synovial fluid neutrophils (**Figure 1D**).

We further analyzed all the quantitative proteins by Gene set enrichment analysis (GSEA) to identify more pathways that are up- or down-regulated in RA synovial fluid neutrophils. Such analyses revealed 10 pathways with positive normalized enrichment scores (NES). Notably, leukocyte degranulation and ROS production had a positive NES, indicating their upregulation in RA-SF neutrophils (**Figure 1E**). Seven pathways were found to have negative NES, further confirming downregulation of megakaryocyte differentiation, cell spreading, and leukocyte adhesion in RA SF neutrophils (**Figure 1F**). Altogether, RA synovial fluid neutrophils displayed a distinct proteomic profile with a strong activation signature, as compared with their blood neutrophils.

### 3.2 Elevated levels of neutrophil-derived proteins in RA synovial fluid

**Figure 2:**
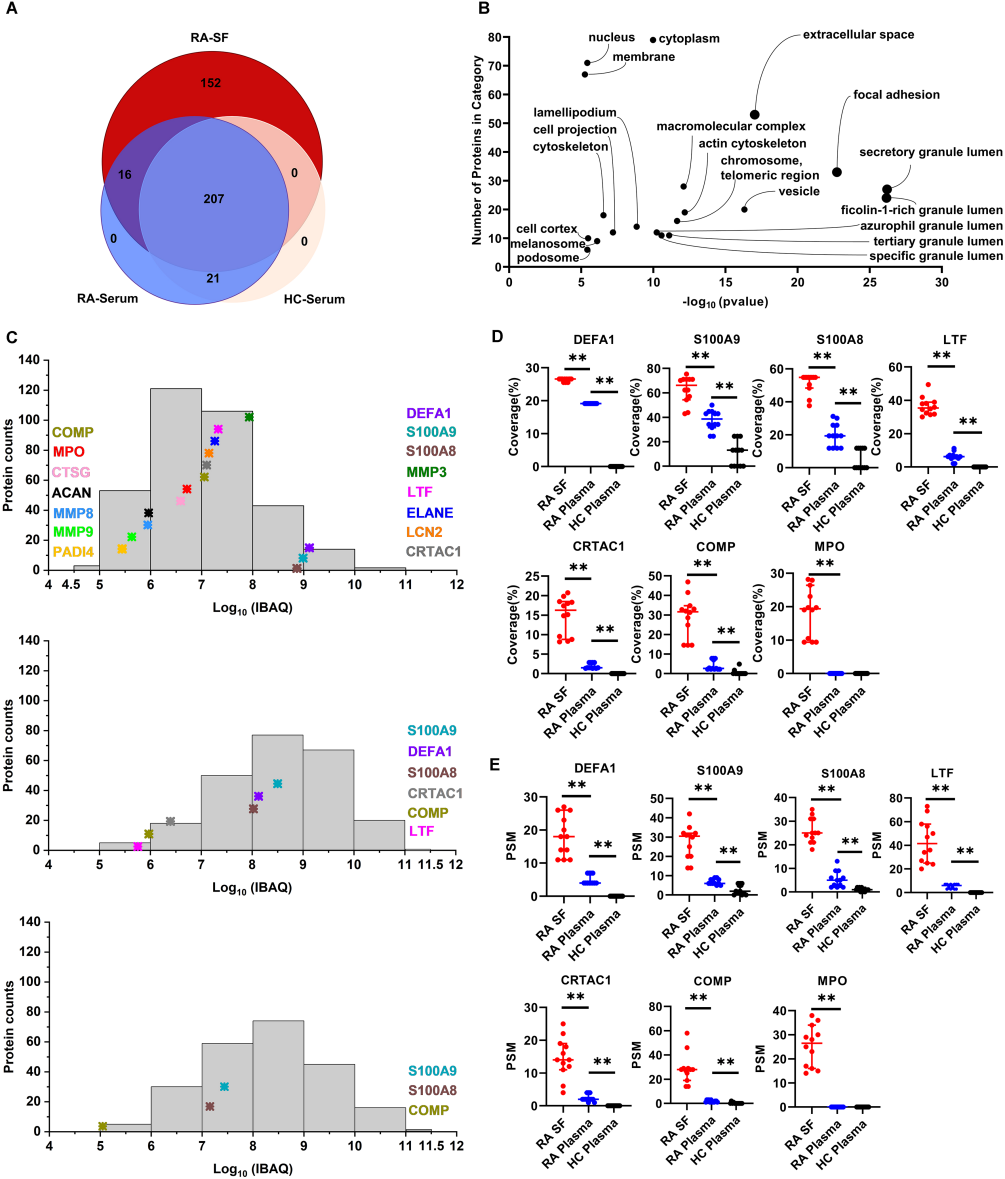
Analysis of neutrophil-derived proteins in RA synovial fluid. **(A)** Comparison of number of proteins identified in RA-SF versus autologous or HC plasma. **(B)** Gene Ontology analysis of cellular component categorization of unique proteins identified in RA-SF samples. The y-axis and x-axis show the number of identified proteins and the False Discovery Rate (FDR) p-value, respectively. **(C)** Comparison of the abundance of neutrophil-derived protein expression in RA-SF and plasma samples from autologous RA and HC. Protein abundance was estimated by log10 values of absolute iBAQ intensities. **(D)** Coverage analysis of significantly upregulated neutrophil proteins in synovial fluid. **(E)** Peptide-spectrum match (PSM) analysis of significantly upregulated neutrophil proteins in synovial fluid. (n = 12/group, **p < 0.01, unpaired T-test). (DEFA1, defensin1; MMP3, stromelysin-1; LTF, lactotransferrin; ELANE, elastase; LCN2, neutrophil gelatinase-associated lipocalin; CRTAC1, cartilage acidic protein 1; COMP, cartilage oligomeric matrix protein; CTSG, neutrophil serine proteases cathepsin G; ACAN, aggrecan core protein; PADI4, protein-arginine deiminase type-4).

Since MS data suggest a strong activation of RA-SF neutrophils with an enhanced degranulation process, we analyzed the extent of activated neutrophil-mediated alterations in the SF composition. To exclude the possibility of contamination from cell components or debris, cell-free SF and blood samples were prepared by cytocentrifugation for 30 min after collecting the samples, as described in materials and methods. In total, more than 3,624 peptides from 396 proteins (each with unique peptides ≥ 2) were determined to be present in RA-SF and plasma samples from the same patients, with healthy individuals as controls. Among them, 152 proteins were exclusively identified in RA-SF samples and 16 proteins were specifically present in both RA-SF and plasma but not in HC plasma samples (**Figure 2A**, **Supplementary Table S4**). Gene ontology (GO) annotation of all the 152 proteins revealed enrichment of subcellular compartments related to neutrophil granules (e.g., azurophilic granules, specific granules, tertiary gelatinase granules, ficolin-1-rich granules), secretory vesicles, focal adhesion and extracellular spaced proteins (**Figure 2B**, **Supplementary Table S5**). Common proteins observed in RA-SF and plasma were mainly associated with acute inflammatory responses (C-reactive protein and prostaglandin-H2 D-isomerase) and neutrophil cytosolic content (neutrophil defensin 1 and lactotransferrin), which reflected the ongoing inflammatory responses in RA patients (**Supplementary Table S5**). While the 21 common proteins found in the plasma samples from RA and HC were macrovesicles and exosome components, encompassing platelet-derived microparticles (PDP) like platelet basic protein, platelet factor 4, thrombospondin-1 and von Willebrand factor, and so on (**Supplementary Table S5**).

Protein abundance in the tested samples was further evaluated by the intensity-based absolute quantification (iBAQ) algorithm. Protein expression in both SF and plasma samples showed a wide range spanning more than seven orders of magnitude (**Figure 2C**). Clearly, RA patients’ SF and, to a lesser extent, RA plasma, but not HC plasma samples, contained elevated levels of neutrophil granule-derived proteins and cytosolic content (**Figure 2C**). In particular, neutrophil cytosolic contents (neutrophil defensin1, S100A9, and S100A8) are among the top 20 most abundant proteins present in RA-SF. In addition, some well-known neutrophil granule-derived proteins (like lactotransferrin, stromelysin-1, elastase, neutrophil gelatinase-associated lipocalin, and MPO) were observed to be significantly present at overabundant levels in RA-SF compared to RA-plasma and HC-plasma (**Figure 2C**). Besides, synovial fluid contained significantly increased levels of matrix metalloproteinases (MMP3, MMP8, and MMP9), and cartilage-specific proteins, including cartilage oligomeric matrix protein, cartilage acidic protein 1, and aggrecan core protein compared to plasma samples from RA or HC (**Figure 2C**). This could be because of the ongoing degradation of cartilage proteins from the cartilage by matrix metalloproteinases and subsequent release *in situ* within the inflamed joints. Moreover, peptide sequence coverage and peptide signal match (PSM) analysis also confirmed the significantly higher level of these neutrophil-deriving proteins and cartilage proteins in synovial fluids compared to plasma samples from RA or HC (**Figures 2D, E**). For example, peptide sequence coverage of MPO (745 aa) reached 30% in a few individual SF samples. However, MPO detection was negligible in the plasma samples from RA and HC by MS. These data further support a robust release of cytoplasmic contents by the infiltrating neutrophils in the SF during inflammation within RA joints.

### 3.3 Increased carbamylation in RA-SF

**Figure 3:**
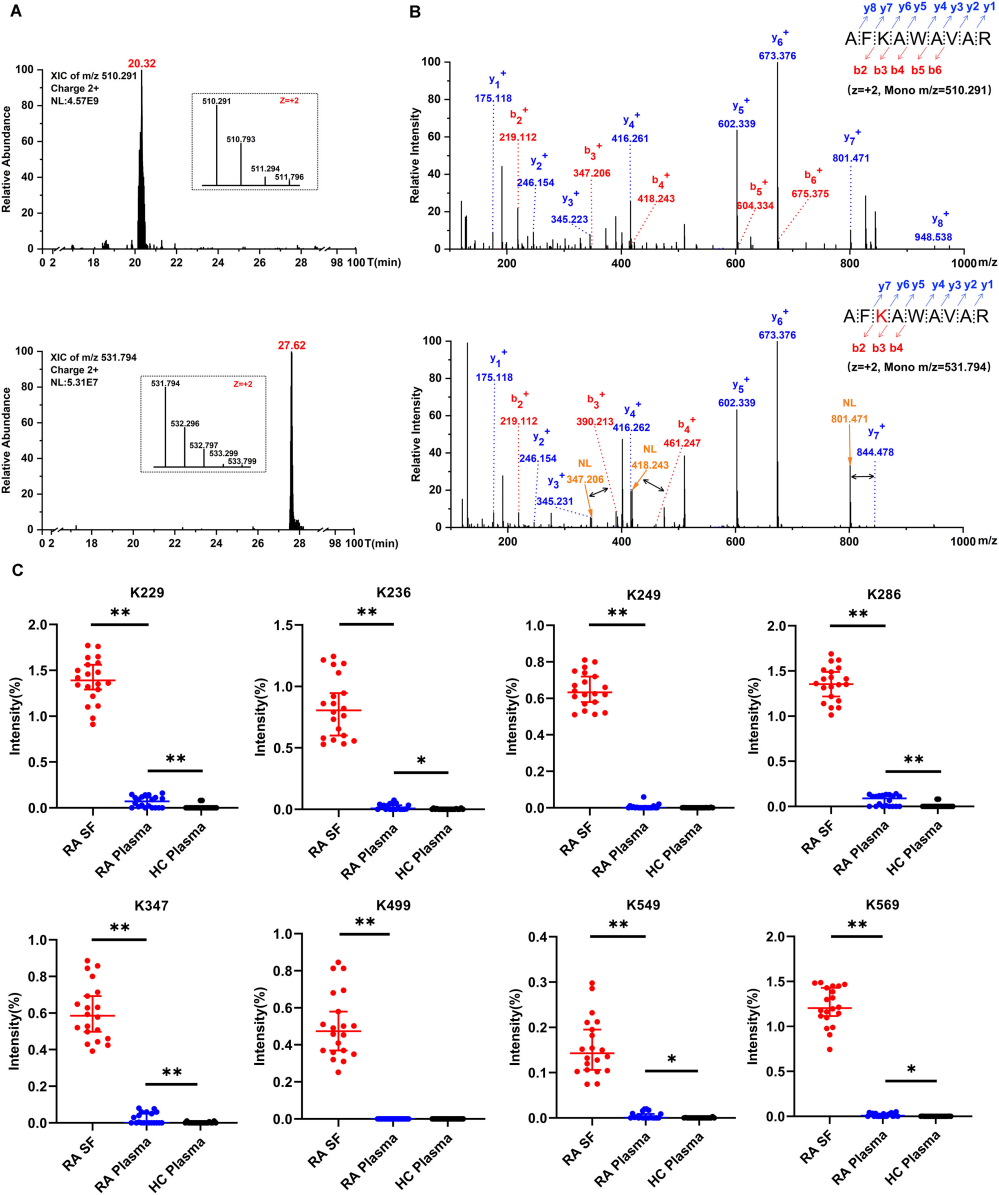
Detection of carbamylated peptides and quantification of albumin carbamylation. **(A)** A typical extracted ion chromatogram of albumin K236 peptide with and without carbamylation. **(B)** MS/MS spectra of albumin K236 peptide containing either unmodified or carbamylated K236 residue. The black arrow depicts specific neutral loss (NL) of isocyanic acid from b3, b4, and y7 fragmented ion; the red K depicts carbamylation. **(C)** Estimate of the relative abundance of albumin carbamylation sites. The percentage was calculated as the intensities of carbamylated peptides divided by the intensities of extracted ion chromatograms of parent peptide, independent of modifications. Values are expressed as median + 95% confidence interval (n = 20, *, p < 0.05, **, p < 0.01).

To analyse the extent of carbamylation, we searched the MS data for lysine-carbamylation and -acetylation in parallel by using MaxQuant software with a 5 ppm tolerance level, which could allow for automatic isotope correction of parent ions and distinguish from ^13^C isotope of lysine-acetylation. In addition, each assigned carbamylation spectrum was manually inspected, and a carbamylation site was considered confident only if the b or y -ion series in a fragmentation spectrum was continuous on both sides of the carbamylated lysine residue. A representative extracted ion chromatogram and MS/MS spectra for carbamylated albumin K236 peptide versus its corresponding native peptide (AF^236^KAWAAVR) are shown in **Figures 3A, B**. This typical carbamylated peptide exhibits delayed retention in its chromatographic behavior, mass addition (+43 Da) on precursor ion (**Figure 3A**), as well as neutral loss (NL) of isocyanic acid from the fragmented ion (**Figure 3B**).

As listed in **Table 1**, 52 carbamylated residues present on 32 different proteins were detected in RA-SF samples. These proteins include previously characterized carbamylated proteins such as albumin, apolipo-A1, and IgG (14, 27, 28). Furthermore, other carbamylated proteins reported as target antigens for anti-CarP antibodies were also detected in RA-SF samples, like α-1-antitrypsin, fibrinogen, fibronectin, haptoglobin, and serotransferrin (1, 13, 29). Most of the carbamylated proteins were identified only in RA-SF samples, except carbamylated albumin K347 or K549, which were detected occasionally in the RA-plasma. Although neutrophil-derived proteins such as defensin1, S100A9, and S100A8 are among the top 20 most abundantly expressed proteins in RA-SF (**Table 1**), carbamylation was not found on these proteins.

**Table 1:** Carbamylation proteins and sites present in RA SF samples.

Accession	Protein Name	Annotated Sequence*	Site
P02763	α-1-acid glycoprotein 1	TYMLAFDVNDEKNWGLSVYADK	K138
P01009	α-1-antitrypsin	FNKPFVFLMIEQNTK	K392
P01023	α-2-macroglobulin	KDNSVHWERPQK	K1177
P02768	Albumin	AACLLPKLDELRDEGK	K205
P02768	Albumin	LDELRDEGKASSAK	K214
P02768	Albumin	CASLQKFGER	K229
P02768	Albumin	AFKAWAVAR	K236
P02768	Albumin	FPKAEFAEVSK	K249
P02768	Albumin	AEFAEVSKLVTDLTK	K257
P02768	Albumin	ADLAKYICENQDSISSK	K286
P02768	Albumin	LKECCEKPLLEK	K305
P02768	Albumin	NYAEAKDVFLGMFLYEYAR	K347
P02768	Albumin	LAKTYETTLEK	K375
P02768	Albumin	VFDEFKPLVEEPQNLIK	K402
P02768	Albumin	QNCELFEQLGEYKFQNALLVR	K426
P02768	Albumin	VTKCCTESLVNR	K499
P02768	Albumin	EFNAETFTFHADICTLSEKER	K543
P02768	Albumin	KQTALVELVK	K549
P02768	Albumin	EQLKAVMDDFAAFVEK	K569
P02768	Albumin	ADDKETCFAEEGK	K588
P02647	Apolipoprotein A-I	QEMSKDLEEVK	K112
P04114	Apolipoprotein B-100	LLKENLCLNLHK	K4349
P02656	Apolipoprotein C-III	GWVTDGFSSLKDYWSTVK	K071
P05090	Apolipoprotein D	KMTVTDQVNCPK	K176
Q96JP9	Cadherin-related family member 1	TMGSPVQSTLISELKQK	K845
P00450	Ceruloplasmin	MYYSAVDPTKDIFTGLIGPMK	K547
P01024	Complement C3	YFKPGMPFDLMVFVTNPDGSPAYR	K365
P15924	Desmoplakin	KIKNDLNLK	K815
P02675	Fibrinogen beta chain	KGGETSEMYLIQPDSSVKPYR	K247
P02679	Fibrinogen gamma chain	AIQLTYNPDESSKPNMIDAATLK	K101
P02751	Fibronectin	TEIDKPSQMQVTDVQDNSISVK	K1544
P00738	Haptoglobin	KQWINKAVGDK	K077
P00739	Haptoglobin-related protein	AVGDKLPECEAVCGKPK	K083
Q14520	Hyaluronan-binding protein 2	GSRQLLDAKVK	K468
P01857	Immunoglobulin heavy constant gamma 1	FNWYVDGVEVHNAKTKPR	K171
P01857	Immunoglobulin heavy constant gamma 1	VVSVLTVLHQDWLNGKEYK	K200
P01857	Immunoglobulin heavy constant gamma 1	VSNKALPAPIEK	K209
A0A0C4DH39	Immunoglobulin heavy variable 1-58	KPGTSVKVSCK	K038
A0A0B4J1Y9	Immunoglobulin heavy variable 3-72	NSLYLQMNSLKTEDTAVYYCAR	K108
P04430	Immunoglobulin kappa variable 1-16	ASQGISNYLAWFQQKPGK	K061
B9A064	Immunoglobulin lambda-like polypeptide 5	AGVETTKPSKQSNNK	K165
Q27J81	Inverted formin-2	ALDELFEAIEQKQR	K889
P13646	Keratin, type I cytoskeletal 13	LASYLEKVR	K121
Q08722	Leukocyte surface antigen CD47	AVEEPLNAFKESK	K314
Q9ULZ9	Matrix metalloproteinase-17	TYFFKDQLYWR	K447
P08590	Myosin light chain 3	TPKCEMKITYGQCGDVLR	K070
P05155	Plasma protease C1 inhibitor	LYHAFSAMKK	K161
P02787	Serotransferrin	HSTIFENLANKADRDQYELLCLDNTR	K236
P02787	Serotransferrin	KPVDEYKDCHLAQVPSHTVVAR	K258
P02787	Serotransferrin	SDNCEDTPEAGYFAIAVVKK	K452
Q8WZ42	Titin	KYEIVADGR	K13390
P02766	Transthyretin	KAADETWEPFASGK	K055

*: Red K denotes carbamylation.

To quantify the frequency of protein carbamylation, we estimated its relative abundance using purified albumin. A pronounced variation in carbamylation frequency was observed between individual lysine sites present on albumin from RA-SF. Highest frequencies of 1.39%, 1.26%, and 1.20% were observed at positions K229, K286, and K569, respectively. The remaining carbamylated residues had less than 1% frequency (**Figure 3C**). Clearly, RA-SF contains increased levels of protein carbamylation at each quantification site of albumin than plasma samples from autologous RA and HC (**Figure 3C**). These findings indicate specific protein carbamylation occurring in the synovial inflammation milieu.

### 3.4 Correlation between MPO activity of synovial neutrophils and *in situ* carbamylation

**Figure 4:**
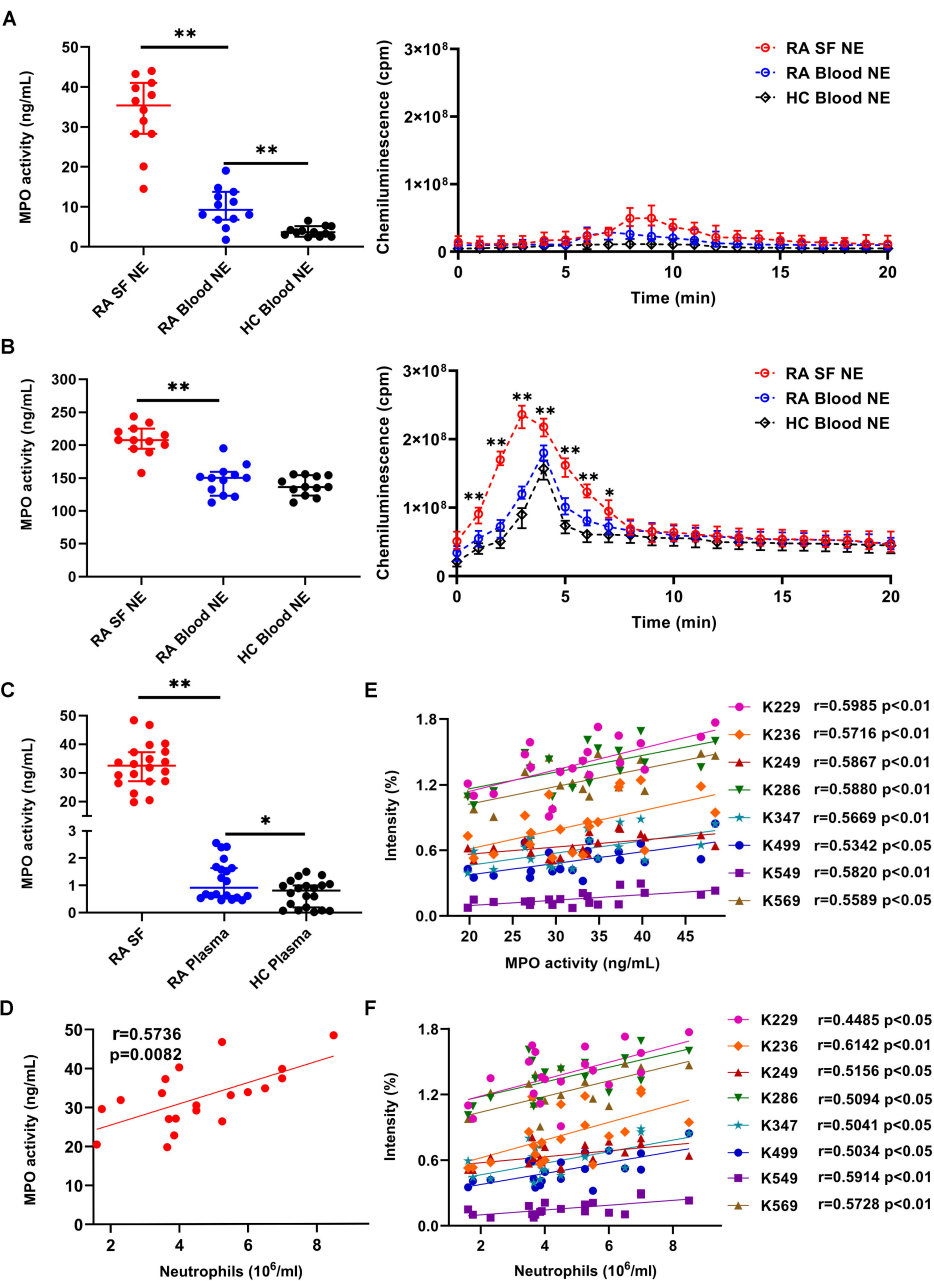
Evaluating the correlation between albumin carbamylation and MPO activity in RA-SF. **(A, B)** Detection of MPO activity and ROS production by different subsets of unstimulated **(A)** or fMLP-stimulated **(B)** neutrophils. Cell supernatants were collected after 2 h of culture (n = 12) and used for analyzing MPO activity. ROS production was analyzed immediately by adding an assay mixture as described in the materials and methods. **(C)** Detection of MPO activity in freshly collected RA-SF and plasma samples from RA and HC. Values in **(A-C)** are expressed as median + 95% confidence interval. **(D-F)** Assessment of correlation between the frequency of albumin carbamylation and MPO activity **(D)**, MPO activity and neutrophil count **(E)**, and the frequency of albumin carbamylation and neutrophil count in RA-SF **(F)**. Spearman correlation coefficients were calculated to detect potential correlations. *, p < 0.05; **, p < 0.01; NE: neutrophils.

Given that MPO and H_2_O_2_ are crucial factors involved in carbamylation, we examined MPO release and H_2_O_2_ production by synovial neutrophils. Under unstimulated conditions, MPO activity was significantly higher in the supernatant from RA-SF and RA blood, but not HC blood neutrophils (**Figure 4A**, left panel). Negligible H_2_O_2_ production was observed under unstimulated condition (**Figure 4A**, right panel). Upon fMLP stimulation, significantly increased levels of MPO release and H_2_O_2_ production were detected in all groups, but highest in neutrophils from RA-SF (**Figure 4B**).

Consistent with our above observations, increased MPO activity in RA-SF were observed by mass-spectrometric analysis. MPO activity was significantly higher (36.1-fold increase) in RA-SF (median 32.5 ng/mL, 95% CI 27.1-37.25 ng/mL) than in RA plasma (0.9 ng/mL, 95% CI 0.6-1.6 ng/mL), while MPO activity was hardly detectable in HC plasma samples (**Figure 4C**). Correlation analysis was done to evaluate for any potential association between the degree of carbamylation in SF and MPO activity or neutrophil counts. The degree of carbamylation, as represented by the frequency of carbamylated albumin, significantly correlated with MPO activity (**Figure 4D**). Similarly, a strong correlation of MPO activity with neutrophil counts in RA-SF was observed (**Figure 4E**). Further data analysis revealed a positive correlation between the degree of albumin carbamylation and neutrophil counts (**Figure 4F**). Concerning whether smoker had more MPO releasing into SF and whether smoker shown high carbamylation degree, unfortunately, in the tested RA samples, only 1 smoker, 2 previous smoker (one year before) and 17 non-smoker were collected, it was thus hardly to investigate associations between smoking, MPO releasing, and carbamylation degree in our current studies (**Supplementary Figures S3**, **S4**). Collectively, these results suggest a significant contribution of SF infiltrating neutrophils and neutrophil-derived MPO to protein carbamylation in SF.

## 4 Discussion

The generation of carbamylated antigens is abundant in neutrophilic inflammation and could be of importance for development and function of autoantibodies in RA. Identification of the origin of carbamylated autoantigens may help to gain more insights into RA pathogenesis.

Here, for the first time at the proteomic level, a unique carbamylation pattern in RA joint localized neutrophils has been demonstrated. RA-SF neutrophils showed an up-regulation of proteins involved in granulocyte activation, degranulation, glucan metabolic process, defense responses, IL-1 production, ROS metabolic processes and, antigen processing and presentation. Proteins associated with megakaryocyte differentiation, leukocyte spreading and adhesion, integrin-mediated signaling pathway, and cell migration were down-regulated. These observations correspond to protein cargo shifts related to the activation of neutrophils as well as their migration from blood to the inflamed joints. Exposure of neutrophils from healthy donor blood samples to RA-SF have been described to delay apoptosis, reduce ROS production, and enhance release of neutrophil extracellular traps (NETs) (18, 30).

Previous flow cytometric studies have reported a presence of hyper-activated neutrophils in RA-SF, with a high expression of activation-associated surface markers, including CD14, CD64 (a.k.a. FcγRI), PD-L1, ICAM-1, CXCR4, HLA-DR or DB, and lower expression of CXCR1 (31–34). Moreover, a recent transcriptomic study revealed decreased expression of genes in RA-SF neutrophils associated with extravasation and migration, increased expression of chemokines, FcγRI, and HLA-II, reduced apoptosis, and increased ROS and NET formation (30). Consistent with these findings, our proteomic data show significantly increased levels of HLA-II proteins, especially HLA-DR and DB molecules in RA-SF neutrophils. It should be noted that HLA-II molecules are generally absent on the surface of blood neutrophils, which could be up-regulated after stimulation (35). Another pronounced up-regulated molecule is the tetraspanin CD63, a specific membrane protein of primary granules, which has a critical role in the sorting and packing of granular proteins in neutrophils (36, 37). Therefore, our proteomic data indicate the level and extent of neutrophil activation in RA-SF.

Our data demonstrate the presence of many neutrophil-derived proteins in RA-SF. Importantly, defensin 1, S100A9, S100A8, lactotransferrin, and elastase were found to be the major proteins observed in the RA-SF neutrophils. This finding is in accordance with the enhanced degranulation and NET formation capacity of RA-SF neutrophils (18, 30, 38) and the presence of highly abundant S100A9, S100A8, lactotransferrin, and elastase proteins in neutrophils (39, 40). Therefore, neutrophil-derived proteins, or NET components (i.e., S100A9/S100A8, elastase, and cell free-DNA) in the plasma and SF of RA patients were suggested as prospective disease activity biomarkers for RA (17, 41–43). The readily detectable neutrophil proteins in SF, and the tissue-destructive effects of neutrophil proteases like elastase and MMP9, suggest an important contribution of neutrophils in driving joint inflammation and destruction in RA patients. Moreover, lactoferrin could serve as an anti-apoptotic molecule and inhibit neutrophil apoptosis (44) and the endogenous damage-associated molecules S100A9/S100A8 could induce leukocyte recruitment and amplify leukocyte activation by binding to TLR4, thus leading to a positive feedback loop during inflammation (45).

In this study, many carbamylated proteins identified in RA-SF were of hematopoietic origin. However, neutrophil-derived carbamylated proteins were hardly detectable. Notably, carbamylated proteins known to be targeted by anti-CarP antibodies including albumin, alpha-1 antitrypsin, and fibrinogen (14, 27, 29, 46), were clearly identified in SF, though other neutrophil proteins (defensing 1, S100A8, and S100A9) constitute a major portion of the SF proteins. A strong association of protein aging with carbamylation reported earlier (47, 48) could be a plausible explanation for the observed less prominent carbamylation of these abundant neutrophil proteins. The short life span of these neutrophils-derived proteins in extracellular environment might have contributed to their low carbamylation status. However, technical limitations posed by mass spectrometers in detecting low level protein modifications in the complex samples could not be ruled out, which requires development of specific enrichment methods for detection of carbamylated proteins in the future.

The neutrophil-derived MPO-H_2_O_2_ system induces carbamylation at inflammation sites (49, 50) through NET formation, which could damage articular joints via carbamylated protein-associated immune complexes (51). However, it should be noted that inhibiting NET formation didn’t substantially interrupt neutrophil-mediated carbamylation, which could be due to protein carbamylation catalyzing function of degranulation process by itself (52). In the present study, a high level of MPO-release and H_2_O_2_ production by RA-SF neutrophils was observed compared to autologous blood neutrophils, indicating a robust release of MPO and H_2_O_2_ into inflammatory joints by SF neutrophils. Moreover, the degree of protein carbamylation, in particular albumin, showed a significant correlation with MPO activity and neutrophil counts in RA-SF. Therefore, we propose MPO-catalyzed carbamylation events are the most likely cause for the presence of carbamylated antigens in the RA joints.

Carbamylation promotes protein antigenicity and carbamylated proteins exhibit strong antigenic properties by inducing T cell activation and antibody production in both RA patients and experimental arthritis models (3, 53–55). Furthermore, carbamylation of proteins exposes antigenic epitopes that can be recognized by ACPAs, due to the similarity to the citrullinated side chain (56). Hence, it is reasonable to speculate that autoantibody and carbamylated protein immune complexes formed in the joints can recruit more neutrophils to the inflammatory foci, triggering a forward-feedback cycle perpetuating RA development.

This is the first study exploring the link between SF infiltrating neutrophils and carbamylated antigen generation in RA. Neutrophils were hyper-activated and increased releasing protein cargo including MPO and ROS, into RA-SF. MPO activity in RA-SF correlated with protein carbamylation. Therefore, based on these results, we speculate that synovial neutrophils could regulate inflammation in RA joints, through enhanced or aberrant carbamylation.

## Data Availability

The datasets presented in this study can be found in online repositories. The names of the repository/repositories and accession number(s) can be found below: http://www.proteomexchange.org/, PXD059136.

## Supplementary material

Supplementary Figure 1 Representative images of Wright-Giemsa stained purified neutrophils from RA-SF and blood. Original magnification x 40.

Supplementary Figure 2 Affinity purification of albumin from synovial fluid and plasma samples. SDS-PAGE followed by Coomassie staining shows different protein fractions collected during the albumin purification method. (1) synovial fluid and plasma samples, (2-3) flow through, (4) albumin fraction. M, denotes molecular weight markers.

Supplementary Figure 3 Measurement of MPO activity in smoker, previous smoker, non-smoker of RA patients and healthy control. Values are expressed as median + 95% confidence interval. Smoker: averagely consumed 12 cigarettes every day, previous smoker: smoke one year before.

Supplementary Figure 4 Analysis of the frequency of albumin carbamylation sites in smoker, previous smoker, non-smoker of RA patients. Smoker: averagely consumed 12 cigarettes every day, previous smoker: smoke one year before.
