# Supplementary figures and images for: Proteomic analysis of infiltrating neutrophils from rheumatoid arthritis synovial fluid and their contribution to protein carbamylation
Source: Front Immunol. 2025 Apr 9;16:1563426. doi: 10.3389/fimmu.2025.1563426 (PMC12014540; doi:10.3389/fimmu.2025.1563426)

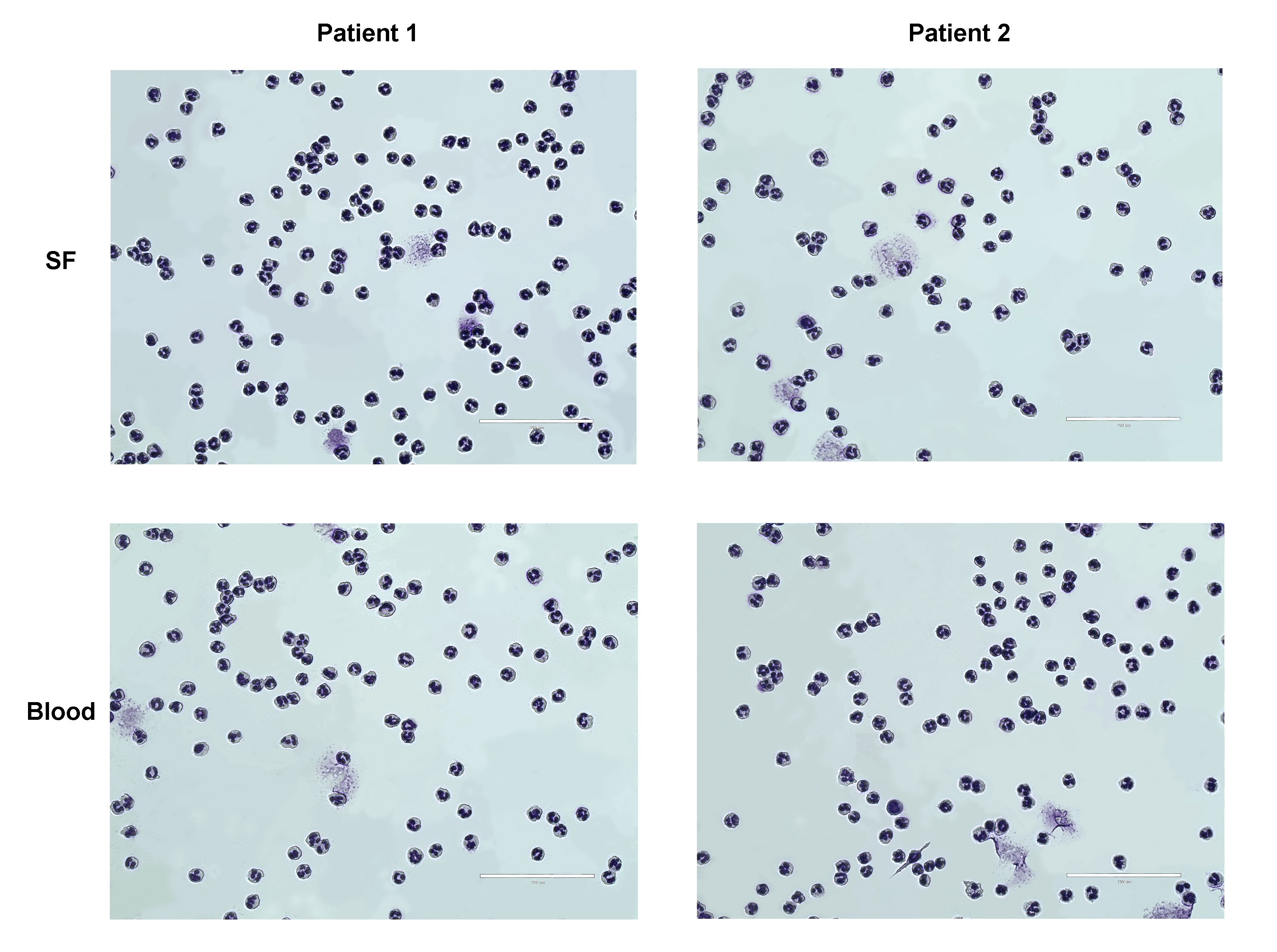

Supplement: Supplementary Figure 1 — Representative images of Wright-Giemsa stained purified neutrophils from RA-SF and blood. Original magnification x 40. [file Image1.jpeg]

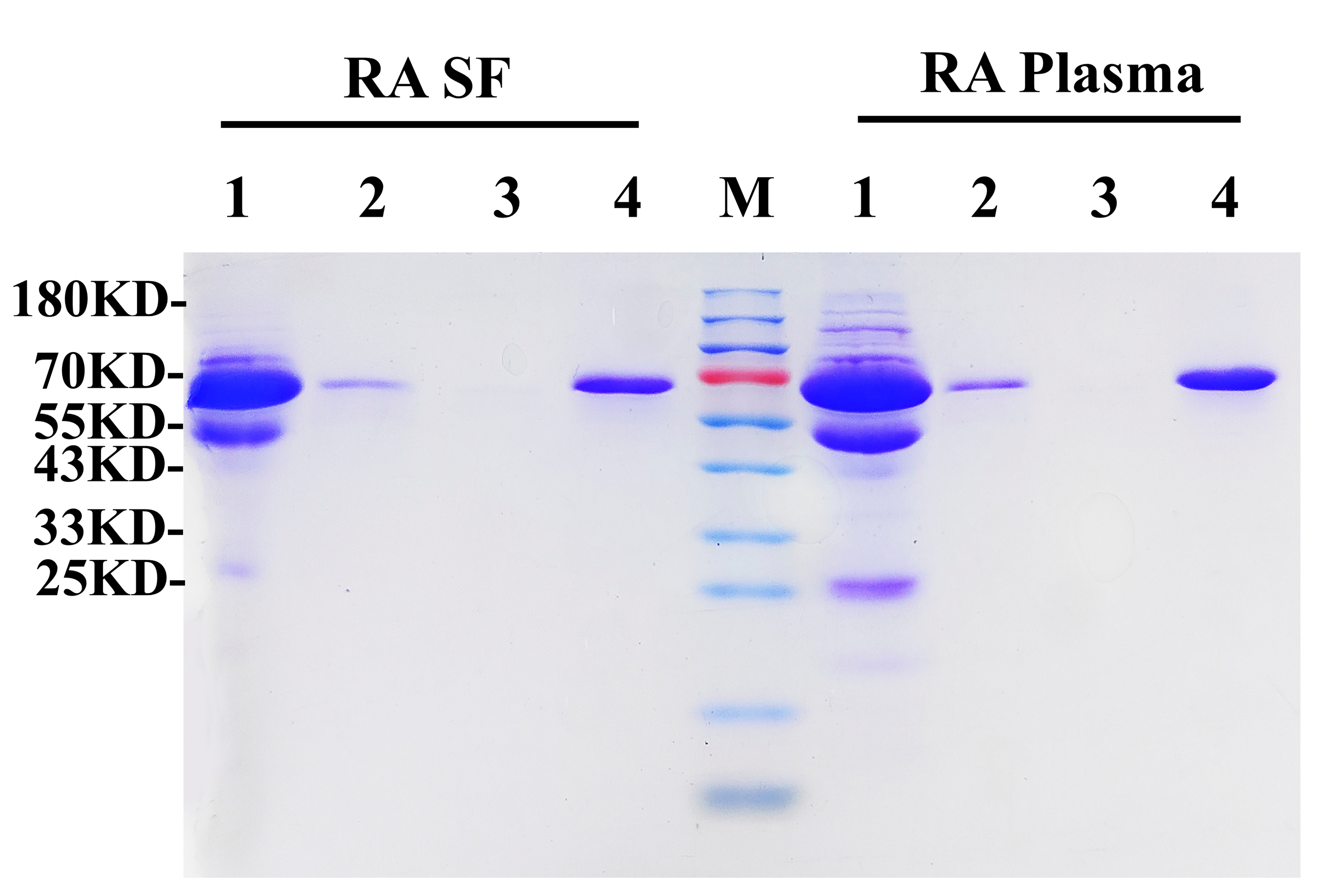

Supplement: Supplementary Figure 2 — Affinity purification of albumin from synovial fluid and plasma samples. SDS-PAGE followed by Coomassie staining shows different protein fractions collected during the albumin purification method. (1) synovial fluid and plasma samples, (2-3) flow through, (4) albumin fraction. M, denotes molecular weight markers. [file Image2.jpeg]

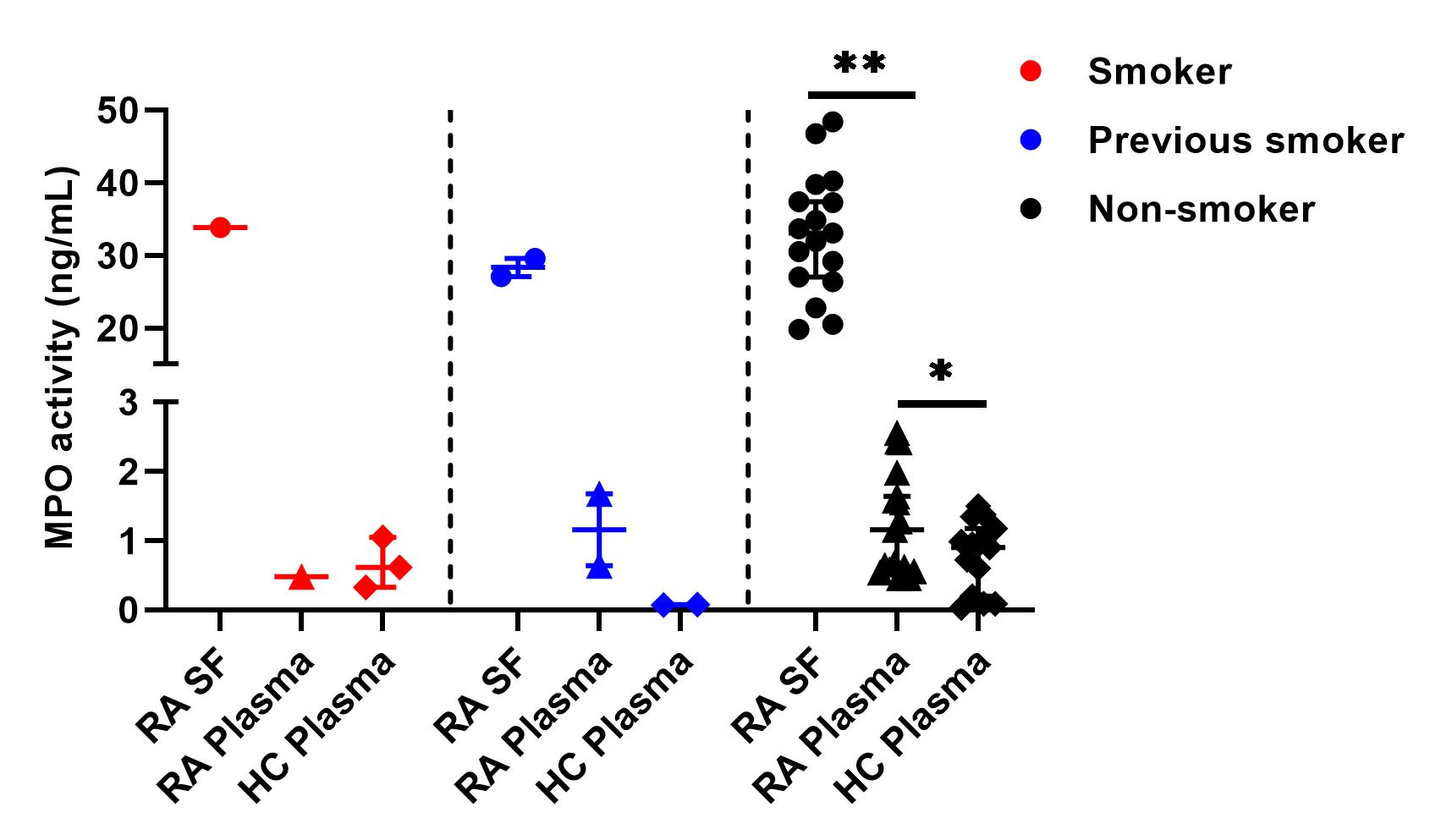

Supplement: Supplementary Figure 3 — Measurement of MPO activity in smoker, previous smoker, non-smoker of RA patients and healthy control. Values are expressed as median + 95% confidence interval. Smoker: averagely consumed 12 cigarettes every day, previous smoker: smoke one year before. [file Image3.jpeg]

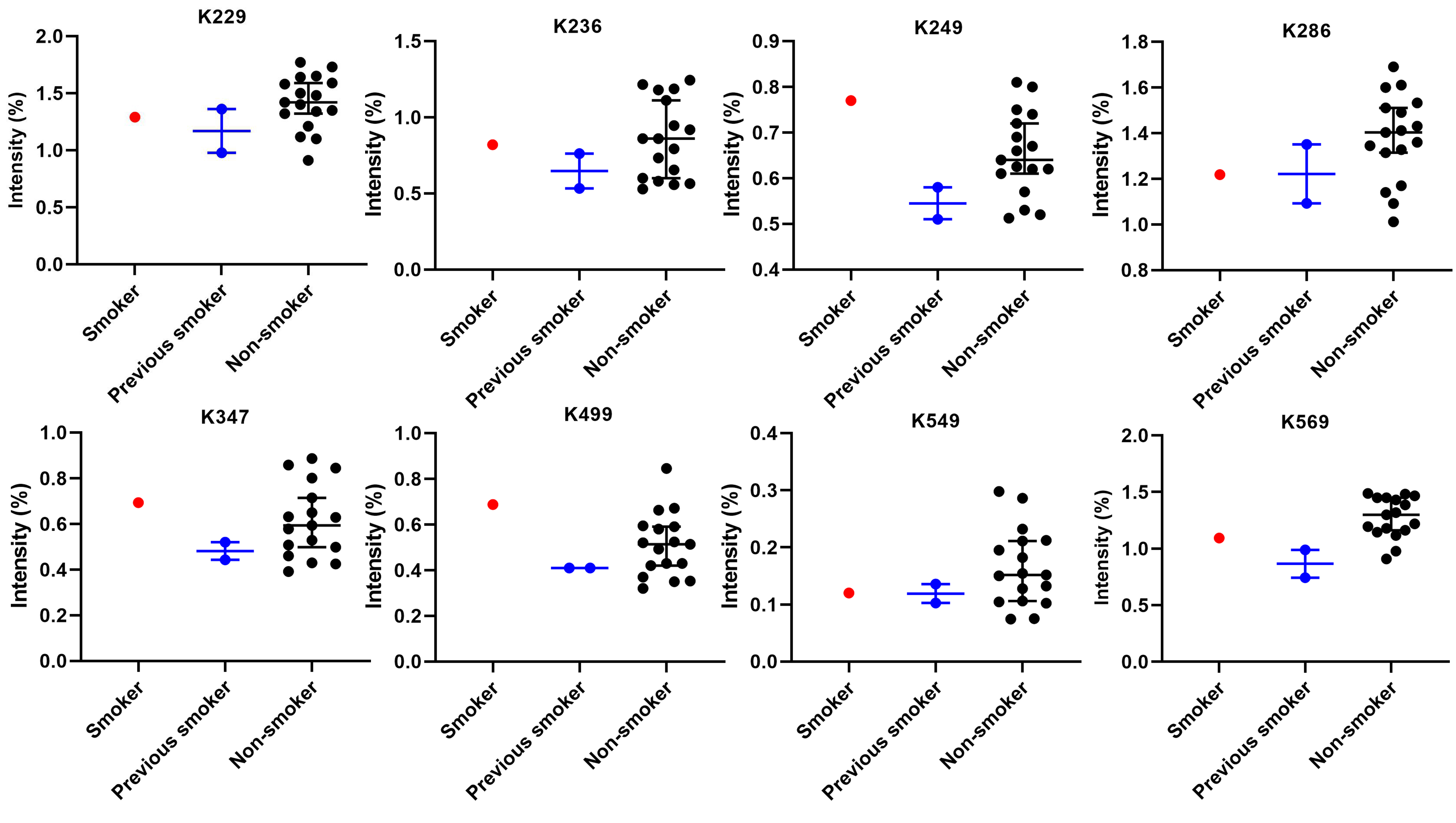

Supplement: Supplementary Figure 4 — Analysis of the frequency of albumin carbamylation sites in smoker, previous smoker, non-smoker of RA patients. Smoker: averagely consumed 12 cigarettes every day, previous smoker: smoke one year before. [file Image4.jpeg]
